# Use of mobile medical teams to fill critical gaps in health service delivery in complex humanitarian settings, 2017-2020: a case study of South Sudan

**DOI:** 10.11604/pamj.supp.2022.42.1.33865

**Published:** 2022-06-09

**Authors:** Diba Dulacha, Otim Patrick Cossy Ramadan, Argata Guracha Guyo, Sylvester Maleghemi, Joseph Francis Wamala, Worri George Wani Gimba, Tony Tombe Wurda, Walla Odra, Chol Thabo Yur, Fredrick Beden Loro, Julu Louis Kenyi Joseph, Emmanuel Timothy Thwol Onak, Stephen Chol Garang Aleu, Kibebu Kinfu Berta, Boniface Ambani Isindu, Olushayo Oluseun Olu

**Affiliations:** 1 The World Health Organization (WHO), Juba, South Sudan

**Keywords:** Vulnerable populations, emergency medical teams, disease outbreaks, public health emergencies, South Sudan, measles, psychosocial care, Rift Valley fever, cholera, rapid response teams

## Abstract

The vulnerable populations in the protracted humanitarian crisis in South Sudan are faced with constrained access to health services and frequent disease outbreaks. Here, we describe the experiences of emergency mobile medical teams (eMMT) assembled by the World Health Organization (WHO) South Sudan to respond to public health emergencies. Interventions: the eMMTs, multidisciplinary teams based at national, state and county levels, are rapidly deployed to conduct rapid assessments, outbreak investigations, and initiate public health response during acute emergencies. The eMMTs were deployed to locations affected by flooding, conflicts, famine, and disease outbreaks. We reviewed records of deployment reports, outreach and campaign registers, and analyzed the key achievements of the eMMTs for 2017 through 2020. Achievements: the eMMTs investigated disease outbreaks including cholera, measles, Rift Valley fever and coronavirus disease (COVID-19) in 13 counties, conducted mobile outreaches in emergency locations in 38 counties (320,988 consultations conducted), trained 550 healthcare workers including rapid response teams, and supported reactive measles vaccination campaigns in seven counties [148,726, (72-125%) under-5-year-old children vaccinated] and reactive oral cholera vaccination campaigns in four counties (355,790 vaccinated). The eMMT is relevant in humanitarian settings and can reduce excess morbidity and mortality and fill gaps that routine health facilities and health partners could not bridge. However, the scope of the services offered needs to be broadened to include mental and psychosocial care and a strategy for ensuring continuity of vaccination services and management of chronic conditions after the mobile outreach is instituted.

## Introduction

The world´s youngest nation, South Sudan, descended into civil war in December 2013, followed by another significant flare-up in 2016 after a failed peace process and has been reeling in the effects of the prolonged conflict ever since [1,2]. The cumulative impact of the conflict and attendant humanitarian crisis has resulted in 8.3 million people in need of humanitarian assistance, 7.2 million people experiencing severe food insecurity, and 2.0 million internally displaced persons (IDP) in 2021 [3-6]. Severe recurrent flooding, acute food insecurity and famine, sub-national violence, mass displacement, disease outbreaks and the COVID-19 pandemic are among the top drivers of humanitarian needs among the communities [7]. The provision of essential health services has been complicated by a health system that is under-developed, under-resourced, and highly dependent on the development and humanitarian partners with limited access to essential health services occasioned by the poor road system, insecurity, and natural disasters like floods [8]. South Sudan has amongst the poorest health indicators owing to poor access to essential services and severe disruption of health service delivery during the decades of civil war. The maternal mortality rate at 1,150 per 100,000 live births is the highest in the world, while the under-5-year-old mortality rate at 98 per 1000 live births is among the highest globally [9]. The routine immunization coverage for vaccine-preventable diseases was estimated at <50% in 2020 with lower coverages in the conflict-affected counties in Jonglei, Unity and Upper Nile, which predisposed the vulnerable population to multiple diseases outbreaks [10,11]. Measles outbreaks were confirmed in 24 out of 47 counties, four UN Protection of Civilians (PoC) sites in 2019 and five counties in 2020 [10,11].

The use of mobile medical teams, usually self-sufficient, multidisciplinary medical teams with sufficient flexibility to deploy rapidly, is a common modality in humanitarian emergencies. Mobile clinics deliver preventive (i.e. immunization, screening, and health education) and curative (i.e. treatment of common morbidities and minor surgeries) services to communities that are unable to access health facilities [12]. The mobile health services are operated with clear referral pathways for services that cannot be rendered by the mobile clinic [12]. Mobile clinics are common and favored in humanitarian crises, although they are limited in coverage, expensive and logistically burdensome, and lack sustainability and continuity for chronic illnesses [12-14].

## Case study

### The rationale for emergency mobile medical team (eMMT)

The vulnerable populations in the humanitarian crisis face frequent disease outbreaks and breakdowns in essential services and require an emergency response to provide timely and essential services to prevent and reduce excess morbidity and mortality [15]. With almost 80% of health services being delivered by health partners, the South Sudan health system can barely provide routine service delivery and respond to emergencies [16]. Health service delivery is fragmented and faces enormous challenges due to limited access as only 44% of the population live within 5km of a health facility [17]. The country experiences frequent humanitarian and public health emergencies that exacerbate the needs of vulnerable communities. The World Health Organization (WHO) South Sudan has a standby emergency mobile medical teams (eMMT) within its emergency program. The eMMT is deployed to verify, investigate, and respond to disease outbreaks and other public health emergencies. The WHO eMMT operates within the framework of the Health Cluster with the frontline Health Cluster partners fulfilling their mandate of providing basic health and essential services including surveillance and response to emergencies in fragile, conflict, and vulnerable settings. WHO provides overall technical backstopping for the health cluster response and strategy, and is a provider of last resort that fills in health response gaps in locations where the frontline partners are either lacking or are overwhelmed based by the scale of the acute crisis and scope of response needs. It is in these settings that the WHO eMMTs are deployed to augment the health cluster response. Here we describe the teams´ experiences, challenges, and usefulness in a complex humanitarian setting in South Sudan and share the lessons learned and recommendations for improving the intervention.

### Description of eMMT

WHO South Sudan established the eMMT in 2016. The intent was to have a readily deployable capacity to conduct assessments, investigations, and institute initial life-saving integrated services during acute emergencies. The eMMTs are WHO personnel engaged on short-term contracts to support response to acute emergencies. The eMMTs comprise epidemiologists, clinicians or doctors, nurses, laboratory specialists, nutritionists, health promotion experts, and public health officers or water, sanitation, and hygiene (WASH) experts. The teams are constituted to ensure they are self-sufficient during their field deployment. For each deployment, the team composition is tailored to the unique response needs of the emergency at hand. Each member of the team plays specific roles and responsibilities on the team. The epidemiologist is the team lead and provides technical leadership during outbreak investigations and rapid assessments, planning and coordination of mobile outreaches, training and vaccination campaigns, and data analysis and report writing. The clinicians and nurses are tasked to undertake clinical care of patients during the outreaches and vaccination activities while the laboratory personnel are charged with collecting, packaging and transporting samples collected from the field. The public health officer and WASH experts are involved in health promotion and messaging, while the nutritionist is tasked to spearhead the nutrition component of the intervention. All the team members support their respective areas during training organized for the local health workers or partners.

There have been two to three eMMTs under WHO South Sudan during different emergencies depending on the response needs. The teams are based in Juba, state or county level. While the teams based in the states or counties are specific for those locations, the team based in Juba functions to support any location that lacks local capacity and requires support. The eMMTs are standby rapidly deployable within 24-48 hours of receiving an alert or a report from the counties, surveillance officers, implementing health partners, or community informers or leaders. The teams receive orientation on planning and preparing for deployments, conducting mobile medical outreaches, emergency vaccination campaigns and capacity building of local health workers upon joining the team. The teams also undergo trainer of trainers training for the Integrated Disease Surveillance and Response (IDSR) to ensure that they are adequately skilled to build local health workers’ capacity to report, detect and investigate alerts or suspected outbreaks, and initiate an appropriate response.

The eMMTs are resourced to undertake their mandates while on field deployments. The materials, tools and support provided are dependent on the intervention planned. The teams are usually equipped with the Ministry of Health (MoH) outpatient registers (used during outreaches), referral forms, and information, education and communication (IEC) materials. In addition, the teams are equipped with emergency health kits (containing essential drugs and supplies required during outreaches), laboratory sample collection kits, water sample collection and testing kits, and first aid supplies. The IDSR, case management and other training materials are also provided to the teams to train local health workers. The operation and logistics support are usually provided through WHO field teams who support securing accommodations and local transportation, while the United Nations Humanitarian Air Services (UNHAS) support transportation from the duty station to response sites. The eMMT interventions are mostly supported through emergency donor-funded projects focused on providing short-term humanitarian aid during acute emergencies.

### Interventions implemented by eMMT

**Figure 1:**
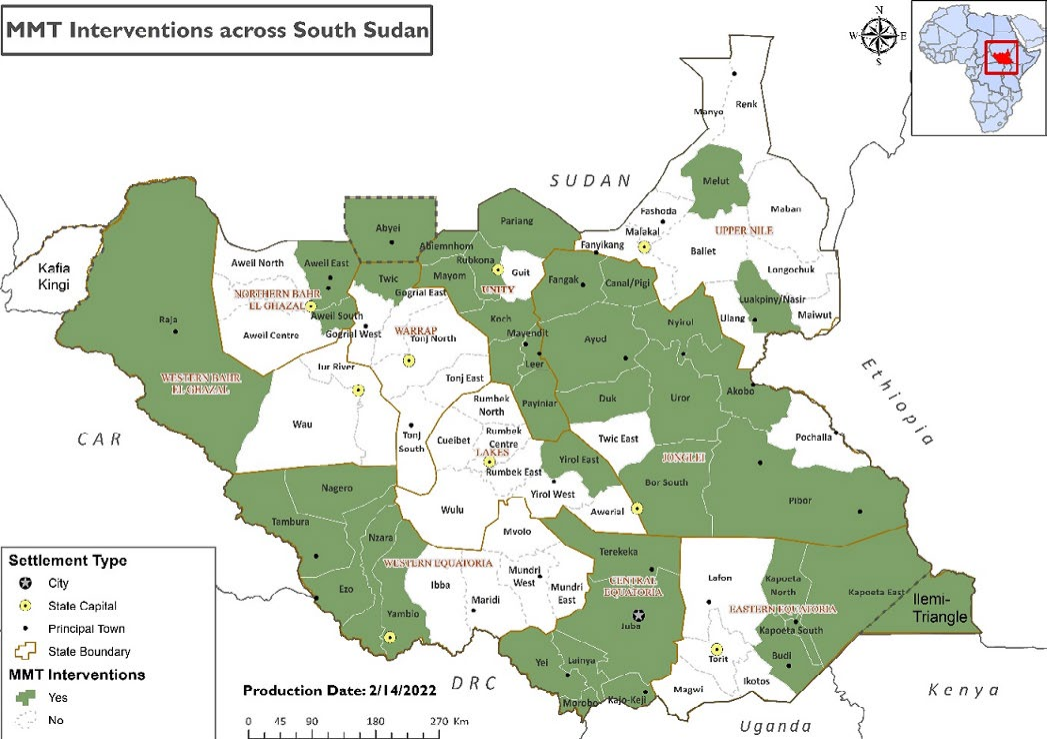
map of South Sudan showing counties where the emergency mobile medical teams´ interventions were implemented, 2017-2020

We deployed eMMTs to locations with acute emergencies where support is required to supplement the existing local capacities. We deployed the eMMTs to respond to acute emergencies such as infectious disease outbreaks, large-scale population displacements, natural disasters like floods, famine and malnutrition, and breakdown in basic health service delivery where there is no other health partner to fill the critical gap. The eMMTs were deployed to over 35 counties across the country during the period 2017-2020 (Figure 1). The Health Cluster, health preparedness and response coordination forum, agrees on the emergency response locations based on the needs assessments and partner presence. WHO mobilized the eMMTs to provide integrated health and nutrition services. The eMMTs were deployed to conflict-affected locations such as Kajo-Keji and Tambura from 2017 to 2020, and flood-affected locations such as Pibor, Akobo and Mayom in 2019 and 2020. During the deployments, the teams select sites for mobile outreaches in consultation with local health leaders and partners to avoid duplication, set up temporary outreach sites and provide services for two to five days. The sites were revisited as per the schedule prepared to ensure adequate coverage of all the locations in need of mobile services. The integrated health and nutrition services provided by the eMMT include preventive (i.e. routine vaccination, antenatal care, health education and promotion), curative (i.e. outpatient consultation, nursing care, minor surgeries, and referral for severe cases) and nutrition (i.e. screening and referral of severely malnourished cases for inpatient care) services. The outreach services were provided as per the clinical package of services by the Health Cluster and the Basic Package of Health and Nutrition Services by the South Sudan Ministry of Health.

**Figure 2:**
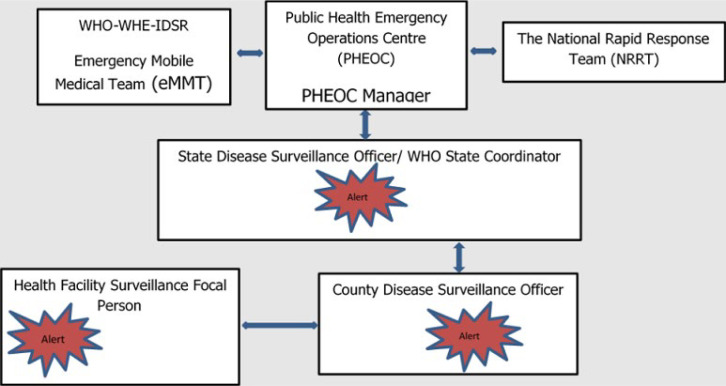
the working relationship between WHO eMMT, PHEOC and NRRT while responding to acute public health events in South Sudan

Secondly, the eMMTs are deployed to conduct alert verification and outbreak investigation. Alerts and suspected disease outbreaks are usually generated through the Early Warning, Alert and Response System (EWARS) and communicated to Public Health Emergency Operation Centre (PHEOC) leadership and WHO by PHEOC officers assigned to monitor alerts. The eMMTs are deployed to conduct outbreak investigations and collect patient and environmental samples for laboratory confirmation. In addition, the eMMT initiated health responses for the affected locations by supporting local health facilities, delivering essential commodities and supplies, and disseminating findings to PHEOC and partners to guide further action. The eMMTs have been deployed to investigate suspected outbreaks of cholera in Pibor and Kapoeta East Counties, yellow fever in Nzara County, and Rift Valley fever in Yirol East County, measles and COVID-19 in several counties. The deployment of eMMTs is done in coordination with the National PHEOC, which is charged with coordinating responses in the country. The eMMTs support the PHEOC through on-job training of the national and state Rapid Response Teams (RRT). The RRTs are the Ministry of Health officers who constitute a critical early detection and response arm of the PHEOC. Figure 2 depicts the working relationship between WHO eMMT, PHEOC, and the RRTs in their mandate to conduct early identification, confirmation of, and response to acute public health events.

Thirdly, we have deployed the eMMTs to implement vaccination activities in locations affected by emergencies. The eMMTs have been deployed during confirmed outbreaks to implement reactive measles and cholera vaccination campaigns and pre-emptive Oral Cholera Vaccination (OCV) campaigns in flood-affected locations. The eMMTs implemented vaccination campaigns in several locations, including Kapoeta South and Pibor. The eMMTs utilized standard MoH vaccination registers, cold chain equipment and supplies, and collaborated with local health workers and partners during the campaigns. The eMMTs have also been deployed to provide training on IDSR, case management of common endemic diseases, infection prevention and control (IPC), and Clinical Management of Rape (CMR) for healthcare workers. The IDSR training that targeted surveillance officers and facility in-charges aims to enhance surveillance and reporting by health facilities and is conducted using the adapted IDSR training modules. The case management training targeted clinicians and nurses, while IPC training was meant for clinical cadres and public health officers. Both the case management and IPC training are based on the treatment and IPC guidelines by the MoH and WHO. All the training utilized customized PowerPoint presentations and case study exercises.

### Data collection and analysis

Data on the outpatient consultation were extracted from the outpatient registers that are completed during mobile medical outreaches. We obtained the information on the number of health workers trained from the attendance sheet. Training and deployment reports were submitted using a standardized training and field report template utilized by the eMMT to document the interventions and achievements. In addition, we extracted the vaccination campaign data from the field reports and the Ministry of Health´s daily tally sheet and summary sheet filled by the vaccinators and team supervisors during the vaccination exercise. The coverage of the vaccination campaigns was calculated using Microsoft Excel as a proportion of the targeted population.

### Achievements eMMT

The eMMT has implemented its intervention in 38 counties spread across nine states, with almost half (42%) of the counties located in Jonglei and Unity States (Figure 1). The interventions implemented ranged from disease outbreak investigations, and implementation of emergency pre-emptive and reactive vaccination campaigns to conducting mobile medical outreaches. Notably, eMMTs have investigated and confirmed rift valley fever outbreaks in 2018. The eMMT investigated measles outbreaks and implemented reactive vaccination campaigns in seven counties during the review period, including Maruwa and Labarab in Pibor, during the widespread measles outbreak in the country in 2019. Labarab and Maruwa are hard-to-reach locations in Pibor Administrative Area, where the eMMT implemented a reactive measles vaccination as the provider of last resort as no partner could access the area. Overall, 148,725 (72-125% coverage) under-5-year-old children were vaccinated in seven locations with confirmed measles outbreaks in 2019 and 2020. Some 355,790 (coverage 51-89% in round 1 and 50-78% in round 2) individuals were reached with oral cholera vaccines in four locations with active cholera outbreaks in 2018. Further, the eMMT implemented a pre-emptive oral cholera vaccination campaign in Bor South, a severe flood county in 2020, and vaccinated 63,280 (88.1%) people in round 1 and 64,137 (89.3%) people in round 2. In 2019 and 2020, the eMMT took the lead in training RRTs and other health workers as part of the country´s preparedness activities for the Ebola Virus Disease (EVD) and COVID-19 pandemic and formed a critical component of the COVID-19 response team after the outbreak was confirmed in the country as members of contact tracers. A summary of some critical achievements is presented in Table 1.

**Table 1:** summary of achievements of eMMT interventions in South Sudan, 2017-2020

Intervention	public health emergency	County	Achievement
Outpatient consultation	OPD and emergencies	38 counties, Bentiu PoC	320988 OPD consultations
Reactive measles vaccination campaign	Measles	Juba, Abyei, Melut, Aweil South, Pibor (Maruwa and Labarab), Bor PoC	148726 vaccinated; coverages achieved: Juba (99%), Abyei (88%), Melut (78%), Aweil South (116%), Pibor-Maruwa and Labarab (72%), Bor PoC (125%),
Reactive oral cholera vaccination campaign	Cholera	Kapoeta East, Kapoeta South, Kapoeta North and Torit,	355790 persons vaccinated; coverages achieved in round 1&2: Kapoeta East [(85058 [(87.7%) vs 75236 (77.6%)], Kapoeta North [(73323 (51.8%) vs 71406 (50.5%)], and Torit [(126895 (79.4%) vs 120452 (75.3%)] Kapoeta South [(70514 (72.7%%) vs 2nd round not done]
Pre-emptive oral cholera campaign	cholera	Bor South	Coverage achieved: 63 280 (88.1%) in round 1 vs 64 137 (89.3%) in round 2
Outbreak investigation	Malaria	Rumbek Centre, Rumbek East, Wulu and Yirol East	Outbreak confirmed; health facilities supplied with emergency kits
	Measles	Juba, Kapoeta East, Pibor,	Outbreak confirmed and responded to
	cholera	Pibor, Kapoeta East and Fangak	The cholera outbreak ruled out
	Rift valley fever	Yirol East	Outbreak confirmed and responded to
	COVID-19	Juba, Torit	Investigated and conducted contacting tracing for the initial COVID-19 cases
	Food poisoning	Bor South, Leer, Kuajok, Aweil	353 patients managed and discharged
Rapid assessments	displacement, floods, conflict, and acute malnutrition	Kajo Keji, Uror, Ayod, Pibor, Renk, Juba, Mayom, Nyirol, Rumbek Centre, Terekeka, Twic East and Nasir	Health facilities assessed and equipped with essential services
Training for health workers (including RRTs)	OPD epidemic-prone diseases	30 counties, Bentiu PoC	550-600 health workers trained
COVID-19 preparedness and response	COVID-19	Juba, Torit	98 RRTs and health workers trained on case investigation and contact tracing
EVD preparedness (training of health workers)	Ebola	Yei, Yambio, Nimule, Juba	65 trained

### Challenges faced by eMMT

The implementation of the mobile medical team strategy has revealed a few important lessons that can be used to strengthen the future approaches for the intervention. First, there is a need for collaboration and coordination with the local administration and partners on the ground for adequate information on insecurity and access challenges critical for planning. Second, the team composition must be reconstituted and adapted based on the nature of the emergency being responded to, assuring adequate capacity within the team to attend the event. A different set of skills and experiences is required to respond to different emergencies adequately. Thirdly, to ensure the needs of the vulnerable populations are addressed comprehensively, it would be prudent to broaden the scope of the services offered by the eMMTs to include psychosocial and mental care. Mental health and psychosocial disorders, including post-traumatic stress syndrome and depression, are prevalent among conflict-affected populations [18]. There is a high need for mental health services during and after conflicts [19]. The effectiveness of delivering community mental health services through mobile health clinic approach has been demonstrated in rural Haiti [20]. Further, there must be a proper referral linkage with local health facilities where the mobile outreaches are being conducted to attend to severe clinical cases requiring an inpatient or specialized service. Transportation and costs of referring these cases from outreach sites to the receiving facilities should be anticipated and planned. Fifth, there is a need for collaboration between eMMTs and local health workers, including community health workers during outreaches and outbreak investigations for on-the-job training and skills transfer with the view of promoting ownership and sustainability of interventions.

Our operations and delivery of essential services have been affected by multiple factors ranging from insecurity to difficulties in delivering essential commodities and personnel on time to where they are required. Unpredictable security situations, inefficient and costly logistics, rugged terrain and poor road and transportation networks, frequent flooding, and lack of continuity of services the mobile teams offer are some of the key challenges faced. The frequent insecurity flare-ups, displacement of the targeted populations, flooding of airstrips, impassable roads, delays in delivery of essential medicines, vaccines, emergency responders, and funds required for planned activities and attacks on health facilities or health personnel have contributed to the mobile missions being postponed, terminated, or being unsuccessful. Many of the locations served by the mobile teams are hard-to-reach areas that caused the teams to trek for long hours through challenging terrains, mud, or flood water or be flown into the area to reach the vulnerable, isolated groups. Some locations lacked a partner or local authority to address the reported emergencies and required WHO to send in the mobile teams as the provider of the last resort.

The lack of continuity of the services offered by the eMMTs is related to the absence of strategies to ensure the continued provision of vaccination services and the management of the chronic conditions initiated during the mobile outreach. The lack of predictability of the visit by the mobile teams may negatively affect the community´s uptake of the services and the ability of the teams to offer certain services. The other notable challenges encountered include weak public health surveillance and poor mobile network coverage in the conflict-affected counties contributing to delays in receiving and responding to reports of public health events and inconsistent capture and reporting of data from the mobile clinics.

## Conclusion

Our experiences illustrated that mobile medical teams are relevant in complex humanitarian settings and can provide basic health services and fill gaps that static health facilities and frontline health cluster partners could not bridge. The eMMTs have demonstrated their importance and potential in averting excess morbidity and mortality associated with such emergencies by promptly taking health services to the most vulnerable and hard-to-reach underserved communities during acute health needs. The usefulness and effectiveness of mobile teams to deliver essential health services to communities that cannot be reached through existing health facilities have been demonstrated elsewhere [20,21]. Despite the challenges in the operating environment, the eMMTs are relatively rapidly deployable and able to respond to many emergencies adequately. However, the scope of the services offered during mobile outreaches lacks critical services for mental and psychosocial disorders. In addition, there is a lack of proper strategy to ensure continuity of services for the patients that would require long-term care and follow up such as patients with chronic conditions like hypertension and asthma. A strategy ensuring children vaccinated during the mobile outreaches receive the subsequent antigens is lacking.

Maximizing the impact of the eMMT strategy requires several actions. First, we recommend expanding the range of services offered by the eMMTs to include psychosocial, gender-based violence (GBV) services and mental health care that is highly beneficial for the conflict-affected populations. Cost-saving strategies and approach should also be incorporated into the intervention to make the intervention more cost-effective. Second, capacity building and collaborating with community health workers, community-based organizations, and local health facilities will ensure the children vaccinated during mobile outreaches receive their age-appropriate subsequent antigens. Third, strengthen the referral network between mobile outreach sites and functional health facilities in the area. Patients with chronic illnesses will need to be linked to health facilities where they will be monitored, and their prescriptions refilled. Fourth, strengthening coordination with local authorities, partners and security apparatus to obtain guidance that will support the planning and execution of activities in a safe environment. Fifth, the adoption of digital health for data capture, reporting, and offering of remote health care services, including training health workers where availability of the internet and power will allow. Finally, the mobile teams should share the data from the mobile clinics with the health facilities serving the catchment populations where they are conducting their outreaches to capture the relevant data by the health facility for their reporting purposes.
